# Two Faces of Macrophages: Training and Tolerance

**DOI:** 10.3390/biomedicines9111596

**Published:** 2021-11-02

**Authors:** Kiran Zubair, Chaelin You, Geunho Kwon, Kyuho Kang

**Affiliations:** Department of Biological Sciences and Biotechnology, Chungbuk National University, Cheongju 28644, Korea; kz.kiranzubair@gmail.com (K.Z.); clyu104@cbnu.ac.kr (C.Y.); geunho93@cbnu.ac.kr (G.K.)

**Keywords:** macrophages, innate immune memory, trained immunity, tolerance, epigenetics, inflammatory diseases, sepsis

## Abstract

Macrophages are present in almost all body tissues. They detect and quickly respond to “environmental signals” in the tissue. Macrophages have been associated with numerous beneficial roles, such as host defense, wound healing, and tissue regeneration; however, they have also been linked to the development of diverse illnesses, particularly cancers and autoimmune disorders. Complex signaling, epigenetic, and metabolic pathways drive macrophage training and tolerance. The induced intracellular program differs depending on the type of initial stimuli and the tissue microenvironment. Due to the essential roles of macrophages in homeostatic and their association with the pathogenesis of inflammatory diseases, recent studies have investigated the molecular mechanisms of macrophage training and tolerance. This review discusses the role of factors involved in macrophage training and tolerance, along with the current studies in human diseases.

## 1. Introduction

**Figure 1:**
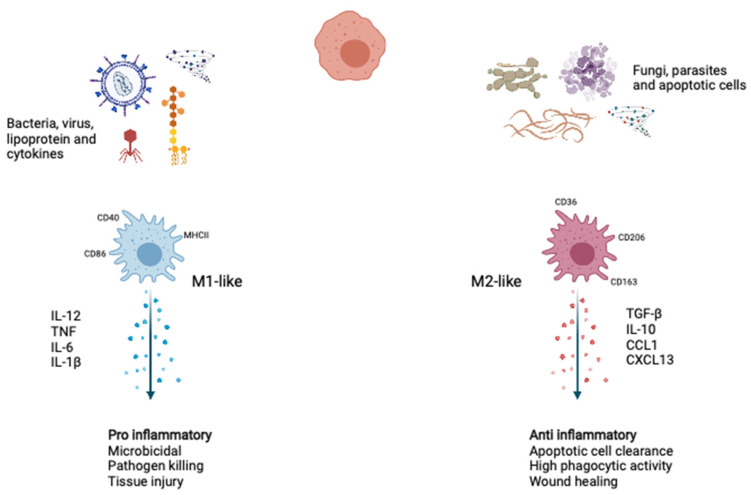
Macrophage polarization. During activation upon stimuli, such as type 1 cytokine, intracellular pathogens, and lipoproteins, the macrophages polarized to the M1-like phenotype show inflammatory and microbicidal. Upon stimuli, such as apoptotic cells and type 2 cytokines, the macrophages polarized to the M2-like phenotype function as anti-inflammatory, wound healing, and apoptotic cell clearance. IL-12, Interleukin-12; TNF, tumor necrosis factor; IL-6, Interleukin-6; IL-1β, Interleukin-1β; TGF-β, tumor growth factor-β; IL-10, Interleukin-10; CCL1, C-C motif chemokine ligand 1; CXCL13, C-X-C motif chemokine ligand 13. Created with BioRender.com.

Macrophages are the body’s first line of defense against pathogens and play a crucial role in innate immunity and have varying maturity and growth potentials and extensively exist as cells with specialized features in various tissues [1,2,3]. Under homeostatic conditions, macrophages adopt phenotypes associated with tissue repair and wound healing. During the acute microbial infection, more myeloid cells are required to replenish consumed innate immune cells. Emergency myelopoiesis increases in the number of myeloid progenitors upon danger signals and inflammatory cytokines. When macrophages encounter pathogens, they express reactive nitrogen and oxygen species and proinflammatory cytokines to aid their antimicrobial and immune-activation functions necessary to kill pathogens. In response to various environmental stimuli and molecular mediators, monocytes differentiate into two representative phenotypes: proinflammatory M1-like macrophages and anti-inflammatory M2-like macrophages (Figure 1).

Since macrophages encounter diverse and dynamic signals temporally and spatially, they show several phenotypes and exhibit functional plasticity. Phenotypic and functional diversity and plasticity are hallmarks of macrophages [4,5,6,7,8]. Recent research suggests that functional reprogramming of macrophages plays a key role in the pathogenesis of chronic inflammatory diseases [9,10,11]. Functional reprogramming of macrophages is associated with two different adaptive programs against secondary allogeneic or heterologous stimuli [12]. In two types of functional reprogramming, secondary responses to subsequent stimuli in innate immune cells can change their ability to respond stronger or lesser than the primary response [13].

**Figure 2:**
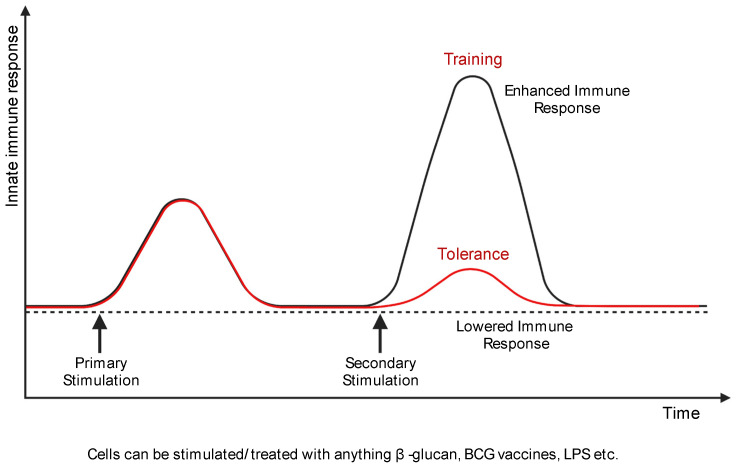
Macrophage training and tolerance. Training of macrophages (black line) is a higher and quicker secondary response upon subsequent stimulation. However, macrophage tolerance (red line) is the attenuated gene expression in response to secondary stimulation. Created with BioRender.com.

Based on these criteria, the dual features of innate immune memory can be defined as training or tolerance [14] (Figure 2). In trained immunity, epigenetic and metabolic reprogramming occur during the activation of immune cells by stimulation [12,14]. As these modifications are maintained, responsiveness to secondary stimulation is enhanced and higher than the primary response [12,14]. Trained macrophages show persistent changes in gene expression and cellular physiology without constant genetic changes like mutation [15]. Environmental stimuli such as LPS, β-glucan, and Bacillus Calmette–Guérin (BCG) can induce trained immunity [13,16]. Trained innate immune cells affect crucial cellular processes like homeostasis and inflammatory responses [17,18].

Tolerance acts opposite to trained immunity. The immune responses of innate immune cells are attenuated after restimulation in the tolerogenic environment [19]. For this reason, the tolerant immune cells show sustaining immunological unresponsiveness to antigens [13,20]. Immune tolerance is also associated with epigenetic changes accompanying innate immune memory [19]. Some studies have demonstrated that epigenetic modifications in the modulation of tolerance and autoimmune disorders [21]. Therefore, strategies to improve or reduce tolerance present new therapeutic possibilities for patients with cancer, inflammatory diseases, and metabolic complications [22].

Rapid activation of inflammatory macrophages is an essential component of host defense. Two main types of innate immune sensors, such as Toll-like receptors (TLRs) and NOD-like receptors (NLRs), provide an immediate response to tissue damage or pathogenic invasion. Upon activation of TLRs or NLRs, macrophages activate the adaptive immune system or tissue repair processes. [23]. Additionally, TLRs or NLRs are present in the damaged tissues of most inflammatory disorders. All TLRs activate NF-κB and AP-1, and some TLRs, such as TLR3 and TLR4, are associated with IRF3 activation. These signaling pathways contribute to the pathogenesis of chronic inflammatory diseases. NLR signaling forms a molecular structural complex called the inflammasome and coordinates with TLR to induce IL-1 and IL-18, which are crucial mediators in most inflammatory disorders. Inflammatory cytokines, such as TNF and IL-1, also mediate acute activation of macrophages [24]. Although the sources of acute stimulation of macrophages are similar to those of trained immunity, prolonged exposure to signals is more associated with the reprogramming of macrophages [12].

Training and tolerance are defined mainly focused on changes in pro-inflammatory genes. Persistent activation of macrophages by low-dose LPS increases histone acetylation and chromatin accessibility, which leads to a training or priming state in response to secondary stimulation. Conversely, prolonged or repeated exposure to high-dose LPS induces a tolerant state, which shows reduced chromatin accessibility and inhibition of transcription factor binding at promoters and enhancers of inflammatory genes, such as TNF and IL-6 [25]. However, the same stimulus can induce two faces of macrophages (both training and tolerance) at the transcriptomic and epigenomic levels. The definition of training or tolerance in macrophages depends on the cluster of genes of interest.

Enhanced inflammatory responses in trained macrophages can lead to pathological tissue damage. For example, trained immunity is an important event explaining the link between infection and cardiovascular disease [13]. Monocytes and macrophages have been identified as essential immune cells for trained immunity to induce vascular inflammation in chronic inflammatory diseases such as atherosclerosis [26]. On the other hand, endotoxin tolerance is closely related to immunoparalysis in sepsis patients. Macrophages have tolerant properties that alter their response to chronic exposure to endotoxins. Tolerant macrophages induce resistance to various proinflammatory cytokines, such as TNF and IL-6, resulting in transcriptional changes, which have many effects on disease [27,28].

In this review, we describe characteristics of macrophages that changed in training and tolerance and provided a better understanding of how the innate immune memory in macrophages functions in chronic inflammatory diseases.

## 2. Training of Macrophages

The innate immune cells of the myeloid lineage are already primed for certain stimuli to respond more strongly with specificity after contacting future stimuli. This feature is known as “trained immunity” which is different from memory in adaptive immunity [29]. Innate immune cells, including monocytes/macrophages and natural killer cells, can recall the incidence of foreign antigen encounters, thus developing an innate immune memory [29].

Trained macrophages can respond more strongly, both quantitatively and qualitatively, than untrained macrophages. Interestingly, metabolic pathways and epigenetic programs can be modulated by specific metabolites, resulting in chromatin remodeling and histone modifications, forming a different trained immunity program [30] (Table 1). Metabolic, epigenetic, and functional reprogramming governs trained immunity in innate immune cells, such as myeloid cells and NK cells [15]. This phenomenon is systematically induced at the bone marrow progenitor level [29,31].

### 2.1. Epigenetic Programming of Trained Macrophages

The alteration of epigenetic plasticity is considered a fundamental factor responsible for macrophage identity and heterogeneity. Dynamic and reversible changes of epigenetic markers at the promoters and enhancers of signal-sensitive genes are critical for the quick reprogramming of macrophage polarization and tailoring the response to a potentially hostile environment [32,33]. Long-term and more persistent epigenetic markers help determine macrophage cell identity [34] leading to the formation of “epigenetic memory”, which affects macrophage responses to the subsequent microbe encounters [35].

DNA-methyltransferase 3 beta contributes to M2 differentiation and phenotypic regulation. In mouse bone marrow-derived macrophages (BMDMs), knockdown of DNMT3B resulted in M2 polarization and prevention of M1 marker genes such as CCL2 and TNF [36]. DNMT1 positively regulates the M1 phenotype by inhibiting SOCS1 in RAW264.7 cells, which is stimulated by LPS, thus inducing *Il6* and *Tnf* expression [37]. Protein arginine methyltransferase 1 (PRMT1) is considered a positive regulator of the M2 phenotype, through the induction of PPAR-γ in IL-4-stimulated mouse peritoneal macrophages [38]. Another histone methyltransferase, SMYD3, is speculated to positively regulate M2 polarization [39]. The H3K27 demethylase JMJD3 (KDM6B) is recognized as an essential regulator of M2 polarization through induction of Irf4, CD206, Arg1, and other M2 markers in IL-4-stimulated [40] and IL-4 + IL-13-stimulated mouse BMDMs [33]. Histone acetylation markers contribute to macrophage phenotypic regulation. H3 acetylation is important for inducing *TNF*, *IL6*, and *IFNα* expression in THP-1 cells, suggesting the significance of H3 acetylation in M1 phenotype [41]. More specifically, H3K9 and H3K14 acetylation of promoters of *Tnf*, *Nos2*, *II6*, and MHC-II in LPS-stimulated mouse microglia are essential for the expression of these genes [42]. Only a subset of genes induces HDAC3 expression for IL-4/STAT6-mediated suppression. The crucial role of IFN in the suppression of M2-related genes is another example of the interaction between epigenetic and transcriptional control [43,44]. IFN-γ-induced macrophage activation is strengthened by a chromatin-based suppression of specific anti-inflammatory pathways in macrophages to attain and maintain an inflammatory state. The functional enhancers associated with M2-like genes, which are enriched for binding transcription factor MAF, are suppressed by IFN-γ via enhancer deactivation [44].

### 2.2. β-Glucan-Induced Training Program

Fungi have β-glucan as a major component in their cell wall, which is commonly used to study trained immunity. The transmembrane C-type lectin receptor, dectin-1, recognizes β-glucan, and thus, the intracellular complex training events are initiated. Many studies have been conducted to evaluate the biological processes induced by β-glucan in monocytes. Immune cell receptors detect antigens or foreign entities, triggering specific-trained immunity by inducing signaling pathways that mediate long-term metabolic and epigenetic adaptation, resulting in a stronger innate immune response after restimulation [45]. Researchers have used a combined transcriptome and metabolomic evaluation approach to discover the three nonredundant metabolic processes (glycolysis, glutaminolysis, and cholesterol synthesis) required for β-glucan-induced monocyte training as evidenced by the inhibition of trained immunity induction whenever one of the pathways is inhibited [46]. The β-glucan-induced training process is directed by crosstalk between any of these primary metabolic pathways. Active glycolysis can then employ the tricarboxylic acid (TCA) cycle to boost mevalonate synthesis, resulting in a positive feedback loop that increases the effect of inducing trained immunity [47]. A whole-genome investigation of H3K4me3 in the context of β-glucan-mediated training in monocytes revealed that many genes involved in the development of atherosclerosis are epigenetically primed for the activation state [45]. As a result, the well-understood process of β-glucan-mediated trained immunity acts as a framework for improving our understanding of the mechanism by which trained immunity contributes to inflammation.

### 2.3. BCG-Induced Training Program

BCG vaccination has also been developed as a paradigm for trained immunity-related investigations because it provides favorable nonspecific benefits. For monocytes, the cytoplasmic pattern recognition receptor, NOD2, plays a significant role [48]. The training program generated via BCG was comparable to that induced by β-glucan. BCG-trained macrophages show a metabolic change similar to β-glucan-trained macrophages, including increased glycolysis, glutaminolysis, and the pentose phosphate pathway [49]. Although BCG-induced training boosts glycolysis and oxidative phosphorylation, it does not always create the traditional Warburg effect seen in β-glucan-trained macrophages [49]. In vivo and in vitro models of trained immunity have been suggested that β-glucan and BCG-induced training require mTOR signaling-mediated cholesterol, glutamine metabolism, and cholesterol synthesis. [47,49]. In terms of epigenetic rewiring, BCG modifies the histone marks H3K4me3, H3K27ac, and H3K9me3 [48,50], which also occur in β-glucan-induced trained immunity. Such epigenetic modifications seem crucial for trained immunity elicited by BCG and are thus involved in controlling inflammatory signaling pathways [48,50]. This epigenetic and metabolic rewiring in BCG-trained macrophages is highly dependent on each other, just like in β-glucan-trained macrophages, where changing one mechanism affects another [49]. Furthermore, it was discovered that the IL-1 pathway is important for BCG-induced trained immunity in human monocytes [50]. Surprisingly, in mice, IFN signaling, rather than IL-1 signaling, is required for BCG-mediated trained macrophages [31].

### 2.4. OxLDL-Induced Training Program

In BCG- and β-glucan-induced trained immunity, several molecular characteristics of oxLDL-trained monocytes and macrophages are found to be significant. First, oxLDL-trained macrophages switch from glycolysis to oxidative phosphorylation, which depends on the mTOR signaling [51,52]. The pharmacological blockade of mTOR activity affects the phenotype of oxLDL-trained macrophages [52]. Fluvastatin inhibits cholesterol production, which blocks oxLDL-mediated trained immunity, demonstrating the overall relevance of the cholesterol metabolic pathway [47]. Finally, oxLDL-primed monocytes experience epigenetic reprogramming, particularly via increased H3K4me3 on genes encoding pro-atherogenic transporters, chemokines, and cytokines [9]. OxLDL-induced trained immunity is abolished when a nonspecific histone methyltransferase inhibitor is used [9]. Furthermore, genetic analyses revealed that the IL-1 pathway, which plays a key role in BCG and β-glucan-induced training immunity, also rules oxLDL-induced monocyte training. The oxLDL-induced training varied from the β-glucan-induced training program. The mTOR-HIF1 axis is a common mechanism for trained immunity triggered by oxLDL or β-glucan. Recently, it was discovered that mTOR signaling in oxLDL-primed monocytes stimulates the generation of reactive oxygen species (ROS), which is necessary for oxLDL-induced trained immunity [52]. In contrast, monocytes trained with β-glucan produce less ROS when mTOR signaling is activated [51]. This intracellular training mechanism regulated via oxLDL, as opposed to a well-known BCG and β-glucan-induced training program, is still unclear. More research that employs epigenomic, metabolic, and transcriptomic techniques, is required to understand this mechanism better.

### 2.5. LPS-Induced Training Program

Tolerance results from a high level of bacterial endotoxin toxin LPS show a weakened immune response to a secondary challenge. However, a subclinical low-dose LPS, as in trained immunity, induces extended innate immunity [53,54,55]. Negative regulators of homeostasis, PI3K and IRAK-M were inhibited for immune tolerance, as demonstrated in previous in vitro studies, along with the induction of IRAK-1 and Toll-interacting protein (Tollip) molecular networks that lead to mild proinflammatory macrophages by LPS-induced priming [53,54].

From a clinical perspective, in atherosclerosis, a risk factor for subclinical endotoxemia, is related to metabolic or chronic disorders [56,57]. The LPS-induced trained immunity worsens atherosclerosis in non-LPS animals after the adoptive transplantation of LPS-primed monocytes [55]. Prolonging activation by LPS subclinical dosage causes low-grade inflammation in atherosclerosis development. Changes in cholesterol metabolism glycolysis and oxidative phosphorylation in trained monocytes are similar to BCG-primed monocytes [58]. But β-glucan primed monocytes or monocytes primed by greater doses of LPS exhibit the Warburg effect. Furthermore, a higher dose of LPS impairs hematopoietic stem/progenitor cells (HSPC) and myelopoiesis [59]. However, β-glucan-mediated trained immunity at the bone marrow level operates properly on HSPCs and myelopoiesis [29].

Another example of LPS-induced training programs is found in the microglia. Microglia are resident immune cells of the central nervous system (CNS) and are also called resident macrophages of the CNS [60]. The primary role of microglia is to ensure synaptic homeostasis and communication with the microenvironment of the CNS. When activated by viruses or bacteria, the somatic cell size of microglia increases, while the shrinkage and concentration of microglia promote their ability to migrate. They express TLRs, which possess the phagocytic capacity and produce inflammatory cytokines [61]. Microglial cells become susceptible to secondary danger signals after priming, thus leading to a more intense immune response [62,63]. Microglia shows an innate immune memory, which is mediated by epigenetic mechanisms [63]. One study compared naïve mice and primed mice with attenuated *Salmonella typhimurium* containing LPS; the primed mice showed greater microglial immune reactivity in response to subsequent stimulation with LPS after four weeks [64]. However, there was no increase in the immune response of microglia from naïve mice. Thus, the epigenetic mechanism underlying the trained immunity of microglia may provide novel insights into the therapeutic strategy of neurodegenerative diseases.

### 2.6. Aldosterone-Induced Training Program

The aldosterone-induced training program via the mineralocorticoid receptor varies from trained immunity induced by β-glucan or BCG [45,46,49]. Aldosterone promotes the accumulation of cholesterol in innate immune cells, leading to the progression of atherosclerosis [65]. In aldosterone-trained macrophages, an increase of the histone mark H3K4me3 in genes related to fatty acid metabolism and proinflammatory cytokines [66] is also seen in β-glucan-, BCG-, and oxLDL-induced trained immunity. Enhanced low-grade inflammation of arterial wall in patients with primary aldosteronism (PA) can be caused by aldosterone instead of hypertensive controls [67]. Therefore, in PA patients, arterial inflammation is present due to the interactions between different immune cells related to atherogenesis [67].

## 3. Tolerance in Innate Immunity

The maintenance of tolerance that inhibits the response to self-antigens such as nucleic acids and cell debris is a key property of M2-like macrophages. Macrophage tolerance can also suppress the tissue damage caused by excessive inflammation [68]. When macrophages are exposed to pathogens repeatedly, the tolerant characteristics show decreased secretion of proinflammatory cytokines and increased expression of anti-inflammatory genes [69].

### 3.1. Epigenetic Mechanisms of Macrophage Tolerance

Complex patterns of epigenetic modifications confer precise control of promoters and enhancers, along with several signaling pathways important for endotoxin tolerance. Various investigations have shown that tolerized macrophages in sepsis models display dynamic epigenetic changes in response to a secondary LPS exposure [70,71]. As a result, TLR-induced genes are classified into different functional groups based on their response to LPS stimulus, each with its own set of epigenetic markers. Upon restimulation, proinflammatory genes are transiently repressed (“tolerized” genes); antimicrobial effectors are not further amplified (“non-tolerized” genes). Tolerized genes have significantly different levels of histone modifications of promoters compared to non-tolerized genes. Tolerized genes retain their baseline promoter state and do not recover the H3K27ac or H3K4me3 marks upon re-exposure to LPS, remaining silent and refractory to activation. Non-tolerized genes, on the other hand, retain the H3K4me3 mark and have their promoters re-acetylated in tolerant macrophages. This result indicates that tolerant macrophages are unable to accumulate H3K27ac at tolerized genes, either due to the lack of proinflammatory activators (e.g., IRF and STATs) or because of the existence of tolerance-inducing transcription factors (e.g., hypoxia-inducible factor 1 alpha, HIF1A) [71].

Different factors can change macrophage tolerance. IFN-γ, which partially restores the production of proinflammatory cytokines in tolerized monocytes and overcomes endotoxin tolerance, adds another layer of epigenetic control at the chromatin level [72]. TLR-induced chromatin remodeling is facilitated by IFN-γ, which recruits ATP-dependent nucleosome remodeling complexes, such as BRG1. It also restores the recruitment of transcription factors and RNA polymerase II to the tolerized genes [71,72].

### 3.2. Endotoxin Tolerance

Endotoxin tolerance (ET) significantly increases the risk of secondary infection and serves as a crucial regulatory mechanism in controlling inflammatory responses [73,74]. When cells exposed to low endotoxin concentrations enter a transient state where they no longer respond to endotoxin is a phenomenon called ET, which is common in both in vitro and in vivo animal models and humans [68,75,76,77,78,79,80,81]. Endotoxin tolerance is a representative example of the complex adaptation of macrophages [82]. Exposure to suboptimal levels of endotoxin (e.g., LPS) resulted in an inability to respond to subsequent LPS challenges in mouse macrophages and human monocytes [76,78,80]. ET in macrophages is defined as hypo-inflammatory status by restimulation with endotoxin such as LPS [83]. Inflammatory cytokines and chemokines are attenuated upon LPS restimulation [76,80,84,85]. The anti-inflammatory cytokines, C-type lectin receptors, negative regulators, and various antimicrobial genes were upregulated in ET [76,80,84,85].

LPS is a ligand that acts primarily through TLR4 and strongly induces inflammatory responses [1,86]. The TLR4 signaling pathway plays an important role in endotoxin resistance by identifying bacterial endotoxins. MyD88 and TRIF mediate the signal transduction of endotoxin into the cell, further promoting the transcription of inflammatory genes [87,88,89,90,91]. Endotoxin tolerance in macrophages is associated with negative regulators of signal transduction, such as IRAK-M and SOCS1 [92,93,94]. Through these negative regulators, macrophages changes to a tolerant phenotype. In addition, the conversion from MyD88-dependent to TRIF-dependent TLR4 pathway promotes anti-inflammatory and tolerant macrophages [95].

### 3.3. TNF Tolerance

TNF is a proinflammatory cytokine and plays a crucial role in regulating inflammatory responses [96,97]. Prolonged exposure to TNF induces a condition similar to endotoxin tolerance in macrophages. TNF-induced tolerance reduces inflammatory cytokines in response to secondary stimulation with LPS [98]. Representative signal transduction that mediates inflammatory activation by TNF occurs through the transcription factor NF-κB [99]. TNF-induced cross-tolerance suppresses transcription of NF-κB target genes. Type I interferon enhances TNF-induced inflammatory responses by chromatin remodeling of inflammatory genes. Transcription factors IRF, ISGF3, and AP-1 in the open chromatin regions may play a pivotal role in TNF-tolerant macrophages [100]. TNF tolerance can help address hyper- or chronic inflammatory diseases such as sepsis, but excessive tolerance can lead to immune paralysis in sepsis [101,102]. However, the abrogation of TNF tolerance can lead to chronic inflammatory diseases such as rheumatoid arthritis and lupus [103]. Therefore, TNF tolerance is considered a therapeutic target for severe chronic inflammatory diseases [104].

## 4. Innate Immune Memory of Macrophages in Human Diseases

Abnormal changes in innate memory programs cause and exacerbate various human diseases related to hyper-inflammation or immunosuppression [105] (Table 2). The enhanced immune responses by training immunity contribute to tissue damage during infection and pathogenesis of atherosclerosis [106,107]. The most crucial changes that occur in trained immunity are metabolic and epigenetic reprogramming of macrophages. Excessive activation in trained macrophages leads to the expression of many inflammatory cytokines, growth factors, and proteases [108].

The study of monocytes in patients with severe atherosclerosis reported that monocytes exhibit a trained immune phenotype by showing an enhanced cytokine production accompanied by epigenetic reprogramming and metabolic rewiring [109]. Recent reports have shown that environmental factors, such as tobacco and microbiome can induce trained immunity related to the pathogenesis of inflammatory diseases [110,111,112]. Alveolar macrophages exposed to long-term tobacco smoke increased IL1A or MCP-1 production, similar to BCG-induced trained macrophages. Promotion of the antimicrobial inflammatory response by these trained macrophages contributes to the exacerbation of chronic obstructive pulmonary disease (COPD) [113]. Thus, the therapeutic strategies targeting metabolic and epigenetic reprogramming in trained monocytes or macrophages can reverse abnormal macrophages in chronic inflammatory diseases [108].

Immune tolerance plays an important role in suppressing immune responses to harmless stimuli such as inhaled allergens and gut microbes [114]. The tolerance of macrophages shows the opposite pattern of trained immunity [115]. Macrophage tolerance reduces immune responses by changes in metabolic activity and histone modifications at promoters and enhancers of inflammatory genes [71]. This persistent inhibition causes aggravation of the disease state by inducing immunoparalysis in sepsis patients [116,117].

### 4.1. Trained Macrophages in Diseases

Macrophages are the key players in chronic inflammatory diseases such as inflammatory bowel disease, rheumatoid arthritis, atherosclerosis, and cancers [118,119,120,121,122]. In chronic inflammation, DAMPs can activate macrophages [123,124,125,126]. Overexpression of proinflammatory cytokines, growth factor chemokines, and metalloproteinases represents the aberrant activation of macrophages [127,128,129].

In patients with RA, the infiltration of macrophages into the synovial cavity contributes to inflammation. Synovial macrophages cause joint damage and inflammation in the acute and chronic phases of RA [128,130]. Metabolic changes, such as increased ATP occur in LPS-stimulated macrophages from RA patients occur due to trained immunity [108,131]. In atherosclerosis, trained immunity induces chronic inflammation [132,133,134]. oxLDL affects atherosclerosis by triggering inflammatory pathways that are required for atherogenesis [135,136,137]. Monocytes exposed to low concentrations of oxLDL in vitro are trained to become pro-atherogenic, with increased foam cell generation capability, synthesis of matrix metalloproteinases, and overexpression of pro-atherogenic chemokines and cytokines because of TLR restimulation [9]. The reduced expression of cholesterol transporters suppresses cellular cholesterol efflux from macrophages primed with super-low dose LPS, increasing the foam cell production in vitro [55]. oxLDL can also generate a trained state by inducing the enrichment of active histone markers in the promoters of *TNF*, *IL6*, and *IL18* genes in monocytes [9]. The examination of circulating monocytes in patients with atherosclerosis confirmed that monocytes displayed a trained phenotype [109]. The hormone aldosterone controls blood pressure and electrolyte balance. Excessive aldosterone has pro-atherogenic effects while blocking the aldosterone pathway reduces the risks to cardiovascular health [65].

When cultured human monocytes with aldosterone for an extended period, they produce proinflammatory cytokines that persist after restimulation. Aldosterone causes an inflammatory state of trained immunity that is associated with primary hyperaldosteronism and inflammatory atherosclerosis mechanisms.

Recently, trained immunity induced through vaccines such as BCG is considered to reduce the risk of SARS-CoV-2 reinfection by increasing the innate immune response. However, whether trained monocytes persist after vaccination has not been confirmed, so additional BCG-related studies on SARS-CoV-2 are needed [14,138]. Alternatively, trained macrophages in oxLDL-induced chronic inflammatory disease can induce uncontrolled hyper-inflammation when exposed to SARS-CoV-2 [139]. By the epigenetic modification of monocytes in convalescent COVID-19 patients, SARS-CoV-2 infection induces macrophage reprogramming [140]. Furthermore, it has been reported that this trained innate reprogramming increases the immunogenicity of the SARS-CoV-2 spike protein and results in a high response to secondary stimuli [141].

### 4.2. Tolerant Macrophages in Diseases

Sepsis is a severe inflammatory response caused by acute infection [142]. The most important factors in sepsis are excessive inflammation and immune paralysis [143,144]. The AMPK-stimulation in the mouse sepsis model reduces hyper-inflammation [145]. Immune paralysis occurs when tolerance induction becomes excessive [19]. In sepsis patients, immunoparalysis persists even after pathogen clearance and contributes to secondary infection and mortality [101]. The disruption of the balance between M1-like and M2-like macrophages promotes the development and exacerbation of sepsis [146]. Continuous stimulation with LPS induces tolerant macrophages that play a role in inducing immunosuppression in sepsis [147]. Many changes, such as desensitization of receptors, secondary activation of negative signaling modules, and induction of anti-inflammatory microRNAs occur in the immune paralysis situation. These changes render macrophages into an LPS tolerance state [148]. During sepsis, monocytes exhibit endotoxin resistance [149]. In the late stage of sepsis, apoptosis of macrophages occurs severely, leading to an increased risk of death due to secondary infection [150,151,152]. Inhibition of the mTOR signaling pathway has been proposed as one of the therapeutic targets that can reduce immune paralysis [45,153]. Treatment with β-glucan and low-dose LPS is also the possible treatment of tolerance in sepsis [30,154,155].

Macrophages are highly abundant in tumors and attract attention as therapeutic targets in cancer immunotherapy [156]. The tumor microenvironment is strongly associated with tumor-associated macrophage (TAM) polarization. TAMs affect resistance to various cancer therapies. Cytokines and chemokines secreted by TAMs suppress the immune system and promote angiogenesis, invasion, and metastasis. A TAM-targeting treatment strategy has not yet been investigated in clinical trials. However, combination therapy targeting TAMs contributes to overcoming the resistance of cancer therapy. Typically, TAMs behave as M2-like macrophages [157]; however, TAMs can express both M1 and M2 markers simultaneously. Damage-associated molecular patterns (DAMPs) produced from dead cells in the tumor microenvironment affect the polarization of macrophages [158]. LPS normally polarizes macrophages to an M1-like phenotype, but repeated LPS stimulation induces tolerance [159]. LPS-tolerant macrophages have an M2-like phenotype that is highly associated with immunosuppression in tumors. Reprogramming macrophages can be a promising approach to address LPS tolerance and immunosuppressive environment in cancers [160]. Macrophage tolerance has the potential to be restored using training immunity. Reprogramming of tolerant macrophages might have a therapeutic effect on human diseases such as sepsis and cancer.

## 5. Conclusions and Future Perspectives

Macrophages play both beneficial and detrimental roles in various diseases, including cancer and autoimmune diseases. As detailed in our review, basic research on macrophage-targeting therapy may contribute to clinical applications. For example, TNF-α and IL-6 receptor inhibitors dramatically improve the prognosis of rheumatoid arthritis and inflammatory bowel disease. Although there are many studies on the role of macrophages in these various chronic inflammatory diseases, the epigenetic and metabolic mechanisms of innate immune memory are not fully understood. Advances in treatment strategies targeting training and tolerance will benefit patients, including those who do not respond well to current therapy.

Trained immunity mediated by β-glucan can lead to a better understanding of the process of inflammation; however, a combination of transcriptomic, metabolomic, and epigenomic studies is required to understand this process entirely. Investigating macrophage tolerance will help understand the mechanisms of the ill effects of trained immunity in several diseases. Myeloid cell-mediated trained immunity can provide a broad-spectrum vaccine with adjuvant development. Dissection of the cellular and molecular mechanisms may facilitate translational research. Further research is needed to focus on a comprehensive understanding of trained immunity and tolerance in macrophages.

## Figures and Tables

**Table 1:** Various components of training programs of macrophages.

Training Programs	Induction of Processes	Epigenetic Modification	Importance/Significance
β-Glucan-induced training	The metabolic process, including glycolysis, glutaminolysis, and cholesterol synthesis pathways.	Histone modifications, such as H3K4 trimethylation and H3K27 acetylation	Regulate inflammation and help the immune system fight diseases such as cancer.
BCG-induced training	Increased glycolysis, glutaminolysis, and the pentose phosphate pathway	Modifies the histone marks H3K4 trimethylation, H3K27 acetylation, and H3K9 trimethylation	Protects against tuberculosis Reduce the risk of SARS-CoV-2 reinfection
OxLDL-induced training	Enhanced foam cell generation capability, synthesis of matrix metalloproteinases, and overexpression of pro-atherogenic chemokines and cytokines because of TLR restimulation	H3K4 trimethylation on genes encoding pro-atherogenic transporters, chemokines, and cytokines.	Triggers inflammatory pathways that are required for atherogenesis Induce uncontrolled hyper-inflammation when exposed to SARS-CoV-2
LPS-induced training	Alter the cholesterol metabolism in these monocytes, and glycolysis and oxidative phosphorylation are similar to BCG-primed monocytes.	Fail to accumulate active histone marks at the promoter and enhancers of genes in the lipid metabolism and phagocytic pathways.	Lower dose can cause trained immunity, while a higher dose can lead to tolerance
Aldosterone-induced training	Induces persistent proinflammatory cytokines	Histone mark H3K4 trimethylation in genes related to fatty acid metabolism and proinflammatory cytokines.	Excessive aldosterone has pro-atherogenic effects while blocking the aldosterone pathway reduces the negative effects on cardiovascular health.

BCG, Bacillus Calmette–Guérin; OxLDL, Oxidized low-density lipoprotein; LPS, Lipopolysaccharide; TLR, Toll-like receptor; SARS-CoV-2, Severe acute respiratory syndrome coronavirus 2.

**Table 2:** Disease linked to abnormal macrophage responses.

Diseases	Macrophage Responses	Mechanisms
Rheumatoid arthritis (RA)	Training	Metabolic changes, such as increased ATP, occur in LPS-stimulated macrophages in patients with RA and result from trained immunity.
Atherosclerosis	Training	Due to TLR restimulation, monocytes exposed to low concentrations of oxLDL in vitro, are trained to become pro-atherogenic.
Chronic obstructive pulmonary disease (COPD)	Training	Training of alveolar macrophages exposed to secondary organ stimulation by cigarette smoke.
Neurodegenerative disease	Training	Microglia exhibit innate immune memory mediated by epigenetic mechanisms. Long-term priming with attenuated Salmonella typhimurium containing LPS reveals greater microglia immune reactivity.
COVID-19	Training	SARS-CoV-2 infection induces macrophage reprogramming, including epigenetic modifications
Cancer	Tolerance	TAMs behave like LPS-tolerant macrophages and M2-like phenotype that is highly associated with tumor immunosuppression.
Sepsis	Tolerance	Prolonged stimulation with LPS also induces resistant macrophages, which contribute to inducing immunosuppression.

COVID-19, Coronavirus disease 2019; ATP, Adenosine triphosphate; LPS, Lipopolysaccharide; TLR, Toll-like receptor; OxLDL, Oxidized low-density lipoprotein; SARS-CoV-2, Severe acute respiratory syndrome coronavirus 2; TAM, Tumor-associated macrophage.

## References

[1] Akira S., Uematsu S., Takeuchi O. (2006). Pathogen recognition and innate immunity. Cell.

[2] Laskin D.L., Pendino K.J. (1995). Macrophages and inflammatory mediators in tissue injury. Annu. Rev. Pharm. Toxicol..

[3] Gordon S. (1995). The macrophage. Bioessays.

[4] Biswas S.K., Mantovani A. (2010). Macrophage plasticity and interaction with lymphocyte subsets: Cancer as a paradigm. Nat. Immunol..

[5] Gordon S., Taylor P.R. (2005). Monocyte and macrophage heterogeneity. Nat. Rev. Immunol..

[6] Murray P.J., Allen J.E., Biswas S.K., Fisher E.A., Gilroy D.W., Goerdt S., Gordon S., Hamilton J.A., Ivashkiv L.B., Lawrence T. (2014). Macrophage activation and polarization: Nomenclature and experimental guidelines. Immunity.

[7] Das A., Sinha M., Datta S., Abas M., Chaffee S., Sen C.K., Roy S. (2015). Monocyte and macrophage plasticity in tissue repair and regeneration. Am. J. Pathol..

[8] Mills C.D. (2012). M1 and M2 Macrophages: Oracles of Health and Disease. Crit. Rev. Immunol..

[9] Bekkering S., Quintin J., Joosten L.A., van der Meer J.W., Netea M.G., Riksen N.P. (2014). Oxidized low-density lipoprotein induces long-term proinflammatory cytokine production and foam cell formation via epigenetic reprogramming of monocytes. Arter. Thromb. Vasc. Biol..

[10] Van der Valk F.M., Bekkering S., Kroon J., Yeang C., Van den Bossche J., van Buul J.D., Ravandi A., Nederveen A.J., Verberne H.J., Scipione C. (2016). Oxidized Phospholipids on Lipoprotein(a) Elicit Arterial Wall Inflammation and an Inflammatory Monocyte Response in Humans. Circulation.

[11] Van Diepen J.A., Thiem K., Stienstra R., Riksen N.P., Tack C.J., Netea M.G. (2016). Diabetes propels the risk for cardiovascular disease: Sweet monocytes becoming aggressive?. Cell. Mol. Life Sci..

[12] Divangahi M., Aaby P., Khader S.A., Barreiro L.B., Bekkering S., Chavakis T., van Crevel R., Curtis N., DiNardo A.R., Dominguez-Andres J. (2021). Trained immunity, tolerance, priming and differentiation: Distinct immunological processes. Nat. Immunol..

[13] Netea M.G., Dominguez-Andres J., Barreiro L.B., Chavakis T., Divangahi M., Fuchs E., Joosten L.A.B., van der Meer J.W.M., Mhlanga M.M., Mulder W.J.M. (2020). Defining trained immunity and its role in health and disease. Nat. Rev. Immunol..

[14] Bekkering S., Dominguez-Andres J., Joosten L.A.B., Riksen N.P., Netea M.G. (2021). Trained Immunity: Reprogramming Innate Immunity in Health and Disease. Annu. Rev. Immunol..

[15] Netea M.G., Joosten L.A., Latz E., Mills K.H., Natoli G., Stunnenberg H.G., O’Neill L.A., Xavier R.J. (2016). Trained immunity: A program of innate immune memory in health and disease. Science.

[16] Kar U.K., Joosten L.A.B. (2020). Training the trainable cells of the immune system and beyond. Nat. Immunol..

[17] Liu B., Liu Q., Yang L., Palaniappan S.K., Bahar I., Thiagarajan P.S., Ding J.L. (2016). Innate immune memory and homeostasis may be conferred through crosstalk between the TLR3 and TLR7 pathways. Sci. Signal..

[18] Netea M.G., Schlitzer A., Placek K., Joosten L.A.B., Schultze J.L. (2019). Innate and Adaptive Immune Memory: An Evolutionary Continuum in the Host’s Response to Pathogens. Cell Host Microbe.

[19] Bauer M., Weis S., Netea M.G., Wetzker R. (2018). Remembering Pathogen Dose: Long-Term Adaptation in Innate Immunity. Trends Immunol..

[20] Sakaguchi S., Yamaguchi T., Nomura T., Ono M. (2008). Regulatory T cells and immune tolerance. Cell.

[21] Mazzone R., Zwergel C., Artico M., Taurone S., Ralli M., Greco A., Mai A. (2019). The emerging role of epigenetics in human autoimmune disorders. Clin. Epigenetics.

[22] Waldmann H. (2010). Tolerance: An overview and perspectives. Nat. Rev. Nephrol..

[23] Fukata M., Vamadevan A.S., Abreu M.T. (2009). Toll-like receptors (TLRs) and Nod-like receptors (NLRs) in inflammatory disorders. Semin. Immunol..

[24] Takeuchi O., Akira S. (2010). Pattern recognition receptors and inflammation. Cell.

[25] Natoli G., Ostuni R. (2019). Adaptation and memory in immune responses. Nat. Immunol..

[26] Zhong C., Yang X., Feng Y., Yu J. (2020). Trained Immunity: An Underlying Driver of Inflammatory Atherosclerosis. Front. Immunol..

[27] Kelly A., Gunaltay S., McEntee C.P., Shuttleworth E.E., Smedley C., Houston S.A., Fenton T.M., Levison S., Mann E.R., Travis M.A. (2018). Human monocytes and macrophages regulate immune tolerance via integrin alphavbeta8-mediated TGFbeta activation. J. Exp. Med..

[28] Butcher S.K., O’Carroll C.E., Wells C.A., Carmody R.J. (2018). Toll-Like Receptors Drive Specific Patterns of Tolerance and Training on Restimulation of Macrophages. Front. Immunol..

[29] Mitroulis I., Ruppova K., Wang B., Chen L.S., Grzybek M., Grinenko T., Eugster A., Troullinaki M., Palladini A., Kourtzelis I. (2018). Modulation of Myelopoiesis Progenitors Is an Integral Component of Trained Immunity. Cell.

[30] Saeed S., Quintin J., Kerstens H.H., Rao N.A., Aghajanirefah A., Matarese F., Cheng S.C., Ratter J., Berentsen K., van der Ent M.A. (2014). Epigenetic programming of monocyte-to-macrophage differentiation and trained innate immunity. Science.

[31] Kaufmann E., Sanz J., Dunn J.L., Khan N., Mendonca L.E., Pacis A., Tzelepis F., Pernet E., Dumaine A., Grenier J.C. (2018). BCG Educates Hematopoietic Stem Cells to Generate Protective Innate Immunity against Tuberculosis. Cell.

[32] Ghisletti S., Barozzi I., Mietton F., Polletti S., De Santa F., Venturini E., Gregory L., Lonie L., Chew A., Wei C.L. (2010). Identification and characterization of enhancers controlling the inflammatory gene expression program in macrophages. Immunity.

[33] Ishii M., Wen H., Corsa C.A., Liu T., Coelho A.L., Allen R.M., Carson W.F.t., Cavassani K.A., Li X., Lukacs N.W. (2009). Epigenetic regulation of the alternatively activated macrophage phenotype. Blood.

[34] Ostuni R., Natoli G. (2011). Transcriptional control of macrophage diversity and specialization. Eur. J. Immunol..

[35] Ostuni R., Piccolo V., Barozzi I., Polletti S., Termanini A., Bonifacio S., Curina A., Prosperini E., Ghisletti S., Natoli G. (2013). Latent enhancers activated by stimulation in differentiated cells. Cell.

[36] Yang X., Wang X., Liu D., Yu L., Xue B., Shi H. (2014). Epigenetic regulation of macrophage polarization by DNA methyltransferase 3b. Mol. Endocrinol..

[37] Cheng C., Huang C., Ma T.T., Bian E.B., He Y., Zhang L., Li J. (2014). SOCS1 hypermethylation mediated by DNMT1 is associated with lipopolysaccharide-induced inflammatory cytokines in macrophages. Toxicol. Lett..

[38] Tikhanovich I., Zhao J., Olson J., Adams A., Taylor R., Bridges B., Marshall L., Roberts B., Weinman S.A. (2017). Protein arginine methyltransferase 1 modulates innate immune responses through regulation of peroxisome proliferator-activated receptor gamma-dependent macrophage differentiation. J. Biol. Chem..

[39] Kittan N.A., Allen R.M., Dhaliwal A., Cavassani K.A., Schaller M., Gallagher K.A., Carson W.F.t., Mukherjee S., Grembecka J., Cierpicki T. (2013). Cytokine induced phenotypic and epigenetic signatures are key to establishing specific macrophage phenotypes. PLoS ONE.

[40] Satoh T., Takeuchi O., Vandenbon A., Yasuda K., Tanaka Y., Kumagai Y., Miyake T., Matsushita K., Okazaki T., Saitoh T. (2010). The Jmjd3-Irf4 axis regulates M2 macrophage polarization and host responses against helminth infection. Nat. Immunol..

[41] Feng D., Sangster-Guity N., Stone R., Korczeniewska J., Mancl M.E., Fitzgerald-Bocarsly P., Barnes B.J. (2010). Differential requirement of histone acetylase and deacetylase activities for IRF5-mediated proinflammatory cytokine expression. J. Immunol..

[42] Chauhan A., Quenum F.Z., Abbas A., Bradley D.S., Nechaev S., Singh B.B., Sharma J., Mishra B.B. (2015). Epigenetic Modulation of Microglial Inflammatory Gene Loci in Helminth-Induced Immune Suppression: Implications for Immune Regulation in Neurocysticercosis. ASN Neuro.

[43] Ivashkiv L.B. (2013). Epigenetic regulation of macrophage polarization and function. Trends Immunol..

[44] Kang K., Park S.H., Chen J., Qiao Y., Giannopoulou E., Berg K., Hanidu A., Li J., Nabozny G., Kang K. (2017). Interferon-gamma Represses M2 Gene Expression in Human Macrophages by Disassembling Enhancers Bound by the Transcription Factor MAF. Immunity.

[45] Cheng S.C., Quintin J., Cramer R.A., Shepardson K.M., Saeed S., Kumar V., Giamarellos-Bourboulis E.J., Martens J.H., Rao N.A., Aghajanirefah A. (2014). mTOR- and HIF-1alpha-mediated aerobic glycolysis as metabolic basis for trained immunity. Science.

[46] Arts R.J., Novakovic B., Ter Horst R., Carvalho A., Bekkering S., Lachmandas E., Rodrigues F., Silvestre R., Cheng S.C., Wang S.Y. (2016). Glutaminolysis and Fumarate Accumulation Integrate Immunometabolic and Epigenetic Programs in Trained Immunity. Cell Metab..

[47] Bekkering S., Arts R.J.W., Novakovic B., Kourtzelis I., van der Heijden C., Li Y., Popa C.D., Ter Horst R., van Tuijl J., Netea-Maier R.T. (2018). Metabolic Induction of Trained Immunity through the Mevalonate Pathway. Cell.

[48] Kleinnijenhuis J., Quintin J., Preijers F., Joosten L.A., Ifrim D.C., Saeed S., Jacobs C., van Loenhout J., de Jong D., Stunnenberg H.G. (2012). Bacille Calmette-Guerin induces NOD2-dependent nonspecific protection from reinfection via epigenetic reprogramming of monocytes. Proc. Natl. Acad. Sci. USA.

[49] Arts R.J.W., Carvalho A., La Rocca C., Palma C., Rodrigues F., Silvestre R., Kleinnijenhuis J., Lachmandas E., Goncalves L.G., Belinha A. (2016). Immunometabolic Pathways in BCG-Induced Trained Immunity. Cell Rep..

[50] Arts R.J.W., Moorlag S., Novakovic B., Li Y., Wang S.Y., Oosting M., Kumar V., Xavier R.J., Wijmenga C., Joosten L.A.B. (2018). BCG Vaccination Protects against Experimental Viral Infection in Humans through the Induction of Cytokines Associated with Trained Immunity. Cell Host Microbe.

[51] Bekkering S., Blok B.A., Joosten L.A., Riksen N.P., van Crevel R., Netea M.G. (2016). In Vitro Experimental Model of Trained Innate Immunity in Human Primary Monocytes. Clin. Vaccine Immunol..

[52] Sohrabi Y., Lagache S.M.M., Schnack L., Godfrey R., Kahles F., Bruemmer D., Waltenberger J., Findeisen H.M. (2018). mTOR-Dependent Oxidative Stress Regulates oxLDL-Induced Trained Innate Immunity in Human Monocytes. Front. Immunol..

[53] Maitra U., Deng H., Glaros T., Baker B., Capelluto D.G., Li Z., Li L. (2012). Molecular mechanisms responsible for the selective and low-grade induction of proinflammatory mediators in murine macrophages by lipopolysaccharide. J. Immunol..

[54] Deng H., Maitra U., Morris M., Li L. (2013). Molecular mechanism responsible for the priming of macrophage activation. J. Biol. Chem..

[55] Geng S., Chen K., Yuan R., Peng L., Maitra U., Diao N., Chen C., Zhang Y., Hu Y., Qi C.F. (2016). The persistence of low-grade inflammatory monocytes contributes to aggravated atherosclerosis. Nat. Commun..

[56] Stoll L.L., Denning G.M., Weintraub N.L. (2004). Potential role of endotoxin as a proinflammatory mediator of atherosclerosis. Arter. Thromb. Vasc. Biol..

[57] Wiesner P., Choi S.H., Almazan F., Benner C., Huang W., Diehl C.J., Gonen A., Butler S., Witztum J.L., Glass C.K. (2010). Low doses of lipopolysaccharide and minimally oxidized low-density lipoprotein cooperatively activate macrophages via nuclear factor kappa B and activator protein-1: Possible mechanism for acceleration of atherosclerosis by subclinical endotoxemia. Circ. Res..

[58] Lachmandas E., Boutens L., Ratter J.M., Hijmans A., Hooiveld G.J., Joosten L.A., Rodenburg R.J., Fransen J.A., Houtkooper R.H., van Crevel R. (2016). Microbial stimulation of different Toll-like receptor signalling pathways induces diverse metabolic programmes in human monocytes. Nat. Microbiol..

[59] Takizawa H., Fritsch K., Kovtonyuk L.V., Saito Y., Yakkala C., Jacobs K., Ahuja A.K., Lopes M., Hausmann A., Hardt W.D. (2017). Pathogen-Induced TLR4-TRIF Innate Immune Signaling in Hematopoietic Stem Cells Promotes Proliferation but Reduces Competitive Fitness. Cell Stem Cell.

[60] Wake H., Fields R.D. (2011). Physiological function of microglia. Neuron. Glia Biol..

[61] Mariani M.M., Kielian T. (2009). Microglia in infectious diseases of the central nervous system. J. Neuroimmune Pharm..

[62] Perry V.H., Holmes C. (2014). Microglial priming in neurodegenerative disease. Nat. Rev. Neurol..

[63] Haley M.J., Brough D., Quintin J., Allan S.M. (2019). Microglial Priming as Trained Immunity in the Brain. Neuroscience.

[64] Puntener U., Booth S.G., Perry V.H., Teeling J.L. (2012). Long-term impact of systemic bacterial infection on the cerebral vasculature and microglia. J. Neuroinflamm..

[65] Van der Heijden C., Deinum J., Joosten L.A.B., Netea M.G., Riksen N.P. (2018). The mineralocorticoid receptor as a modulator of innate immunity and atherosclerosis. Cardiovasc. Res..

[66] Yao Y., Jeyanathan M., Haddadi S., Barra N.G., Vaseghi-Shanjani M., Damjanovic D., Lai R., Afkhami S., Chen Y., Dvorkin-Gheva A. (2018). Induction of Autonomous Memory Alveolar Macrophages Requires T Cell Help and Is Critical to Trained Immunity. Cell.

[67] Van der Heijden C., Smeets E.M.M., Aarntzen E., Noz M.P., Monajemi H., Kersten S., Kaffa C., Hoischen A., Deinum J., Joosten L.A.B. (2020). Arterial Wall Inflammation and Increased Hematopoietic Activity in Patients With Primary Aldosteronism. J. Clin. Endocrinol. Metab..

[68] Cavaillon J.M., Adib-Conquy M. (2006). Bench-to-bedside review: Endotoxin tolerance as a model of leukocyte reprogramming in sepsis. Crit. Care.

[69] Biswas S.K., Lopez-Collazo E. (2009). Endotoxin tolerance: New mechanisms, molecules and clinical significance. Trends Immunol.

[70] Shalova I.N., Lim J.Y., Chittezhath M., Zinkernagel A.S., Beasley F., Hernandez-Jimenez E., Toledano V., Cubillos-Zapata C., Rapisarda A., Chen J. (2015). Human monocytes undergo functional re-programming during sepsis mediated by hypoxia-inducible factor-1alpha. Immunity.

[71] Novakovic B., Habibi E., Wang S.Y., Arts R.J.W., Davar R., Megchelenbrink W., Kim B., Kuznetsova T., Kox M., Zwaag J. (2016). beta-Glucan Reverses the Epigenetic State of LPS-Induced Immunological Tolerance. Cell.

[72] Chen J., Ivashkiv L.B. (2010). IFN-gamma abrogates endotoxin tolerance by facilitating Toll-like receptor-induced chromatin remodeling. Proc. Natl. Acad. Sci. USA.

[73] Rodriguez R.M., Suarez-Alvarez B., Lopez-Larrea C. (2019). Therapeutic Epigenetic Reprogramming of Trained Immunity in Myeloid Cells. Trends Immunol..

[74] Zhou M., Aziz M., Denning N.L., Yen H.T., Ma G., Wang P. (2020). Extracellular CIRP induces macrophage endotoxin tolerance through IL-6R-mediated STAT3 activation. JCI Insight.

[75] Biswas S.K., Tergaonkar V. (2007). Myeloid differentiation factor 88-independent Toll-like receptor pathway: Sustaining inflammation or promoting tolerance?. Int. J. Biochem. Cell Biol..

[76] Del Fresno C., Garcia-Rio F., Gomez-Pina V., Soares-Schanoski A., Fernandez-Ruiz I., Jurado T., Kajiji T., Shu C., Marin E., Gutierrez del Arroyo A. (2009). Potent phagocytic activity with impaired antigen presentation identifying lipopolysaccharide-tolerant human monocytes: Demonstration in isolated monocytes from cystic fibrosis patients. J. Immunol..

[77] Dobrovolskaia M.A., Medvedev A.E., Thomas K.E., Cuesta N., Toshchakov V., Ren T., Cody M.J., Michalek S.M., Rice N.R., Vogel S.N. (2003). Induction of in vitro reprogramming by Toll-like receptor (TLR)2 and TLR4 agonists in murine macrophages: Effects of TLR “homotolerance” versus “heterotolerance” on NF-kappa B signaling pathway components. J. Immunol..

[78] Dobrovolskaia M.A., Vogel S.N. (2002). Toll receptors, CD14, and macrophage activation and deactivation by LPS. Microbes Infect..

[79] Saugel B., Wagner J.Y., Reuter D.A. (2015). Haemodynamic monitoring: The inseparable relation of accuracy and trending. Br. J. Anaesth..

[80] Foster S.L., Hargreaves D.C., Medzhitov R. (2007). Gene-specific control of inflammation by TLR-induced chromatin modifications. Nature.

[81] Medvedev A.E., Kopydlowski K.M., Vogel S.N. (2000). Inhibition of lipopolysaccharide-induced signal transduction in endotoxin-tolerized mouse macrophages: Dysregulation of cytokine, chemokine, and toll-like receptor 2 and 4 gene expression. J. Immunol..

[82] Fu Y., Glaros T., Zhu M., Wang P., Wu Z., Tyson J.J., Li L., Xing J. (2012). Network topologies and dynamics leading to endotoxin tolerance and priming in innate immune cells. PLoS Comput. Biol..

[83] Nomura F., Akashi S., Sakao Y., Sato S., Kawai T., Matsumoto M., Nakanishi K., Kimoto M., Miyake K., Takeda K. (2000). Cutting edge: Endotoxin tolerance in mouse peritoneal macrophages correlates with down-regulation of surface toll-like receptor 4 expression. J. Immunol..

[84] Draisma A., Pickkers P., Bouw M.P., van der Hoeven J.G. (2009). Development of endotoxin tolerance in humans in vivo. Crit. Care Med..

[85] Mages J., Dietrich H., Lang R. (2007). A genome-wide analysis of LPS tolerance in macrophages. Immunobiology.

[86] Kawai T., Akira S. (2010). The role of pattern-recognition receptors in innate immunity: Update on Toll-like receptors. Nat. Immunol..

[87] O’Neill L.A., Dunne A., Edjeback M., Gray P., Jefferies C., Wietek C. (2003). Mal and MyD88: Adapter proteins involved in signal transduction by Toll-like receptors. J. Endotoxin Res..

[88] Funami K., Matsumoto M., Oshiumi H., Inagaki F., Seya T. (2017). Functional interfaces between TICAM-2/TRAM and TICAM-1/TRIF in TLR4 signaling. Biochem. Soc. Trans..

[89] Bohannon J.K., Hernandez A., Enkhbaatar P., Adams W.L., Sherwood E.R. (2013). The immunobiology of toll-like receptor 4 agonists: From endotoxin tolerance to immunoadjuvants. Shock.

[90] Luu K., Greenhill C.J., Majoros A., Decker T., Jenkins B.J., Mansell A. (2014). STAT1 plays a role in TLR signal transduction and inflammatory responses. Immunol. Cell Biol..

[91] Xia M.Z., Liang Y.L., Wang H., Chen X., Huang Y.Y., Zhang Z.H., Chen Y.H., Zhang C., Zhao M., Xu D.X. (2012). Melatonin modulates TLR4-mediated inflammatory genes through MyD88- and TRIF-dependent signaling pathways in lipopolysaccharide-stimulated RAW264.7 cells. J. Pineal Res..

[92] Van‘t Veer C., van den Pangaart P.S., van Zoelen M.A., de Kruif M., Birjmohun R.S., Stroes E.S., de Vos A.F., van der Poll T. (2007). Induction of IRAK-M is associated with lipopolysaccharide tolerance in a human endotoxemia model. J. Immunol..

[93] Nakagawa R., Naka T., Tsutsui H., Fujimoto M., Kimura A., Abe T., Seki E., Sato S., Takeuchi O., Takeda K. (2002). SOCS-1 participates in negative regulation of LPS responses. Immunity.

[94] Liu D., Cao S., Zhou Y., Xiong Y. (2019). Recent advances in endotoxin tolerance. J. Cell. Biochem..

[95] Wang N., Liang H., Zen K. (2014). Molecular mechanisms that influence the macrophage m1-m2 polarization balance. Front. Immunol..

[96] Bradley J.R. (2008). TNF-mediated inflammatory disease. J. Pathol..

[97] Brenner D., Blaser H., Mak T.W. (2015). Regulation of tumour necrosis factor signalling: Live or let die. Nat. Rev. Immunol..

[98] Park S.H., Park-Min K.H., Chen J., Hu X., Ivashkiv L.B. (2011). Tumor necrosis factor induces GSK3 kinase-mediated cross-tolerance to endotoxin in macrophages. Nat. Immunol..

[99] Kalliolias G.D., Ivashkiv L.B. (2016). TNF biology, pathogenic mechanisms and emerging therapeutic strategies. Nat. Rev. Rheumatol..

[100] Park S.H., Kang K., Giannopoulou E., Qiao Y., Kang K., Kim G., Park-Min K.H., Ivashkiv L.B. (2017). Type I interferons and the cytokine TNF cooperatively reprogram the macrophage epigenome to promote inflammatory activation. Nat. Immunol..

[101] Hotchkiss R.S., Monneret G., Payen D. (2013). Sepsis-induced immunosuppression: From cellular dysfunctions to immunotherapy. Nat. Rev. Immunol..

[102] Hotchkiss R.S., Moldawer L.L., Opal S.M., Reinhart K., Turnbull I.R., Vincent J.L. (2016). Sepsis and septic shock. Nat. Rev. Dis. Primers.

[103] Firestein G.S., McInnes I.B. (2017). Immunopathogenesis of Rheumatoid Arthritis. Immunity.

[104] Huber R., Bikker R., Welz B., Christmann M., Brand K. (2017). TNF Tolerance in Monocytes and Macrophages: Characteristics and Molecular Mechanisms. J. Immunol. Res..

[105] Mulder W.J.M., Ochando J., Joosten L.A.B., Fayad Z.A., Netea M.G. (2019). Therapeutic targeting of trained immunity. Nat. Rev. Drug Discov..

[106] Musher D.M., Abers M.S., Corrales-Medina V.F. (2019). Acute Infection and Myocardial Infarction. Reply. N. Engl. J. Med..

[107] Leentjens J., Bekkering S., Joosten L.A.B., Netea M.G., Burgner D.P., Riksen N.P. (2018). Trained Innate Immunity as a Novel Mechanism Linking Infection and the Development of Atherosclerosis. Circ. Res..

[108] Municio C., Criado G. (2020). Therapies Targeting Trained Immune Cells in Inflammatory and Autoimmune Diseases. Front. Immunol..

[109] Bekkering S., van den Munckhof I., Nielen T., Lamfers E., Dinarello C., Rutten J., de Graaf J., Joosten L.A., Netea M.G., Gomes M.E. (2016). Innate immune cell activation and epigenetic remodeling in symptomatic and asymptomatic atherosclerosis in humans in vivo. Atherosclerosis.

[110] Chen G.Y., Nunez G. (2010). Sterile inflammation: Sensing and reacting to damage. Nat. Rev. Immunol..

[111] Brasacchio D., Okabe J., Tikellis C., Balcerczyk A., George P., Baker E.K., Calkin A.C., Brownlee M., Cooper M.E., El-Osta A. (2009). Hyperglycemia induces a dynamic cooperativity of histone methylase and demethylase enzymes associated with gene-activating epigenetic marks that coexist on the lysine tail. Diabetes.

[112] Christ A., Gunther P., Lauterbach M.A.R., Duewell P., Biswas D., Pelka K., Scholz C.J., Oosting M., Haendler K., Bassler K. (2018). Western Diet Triggers NLRP3-Dependent Innate Immune Reprogramming. Cell.

[113] McGrath J.J.C., Stampfli M.R. (2018). The immune system as a victim and aggressor in chronic obstructive pulmonary disease. J. Thorac. Dis..

[114] Medzhitov R., Schneider D.S., Soares M.P. (2012). Disease tolerance as a defense strategy. Science.

[115] Dominguez-Andres J., Netea M.G. (2019). Long-term reprogramming of the innate immune system. J. Leukoc. Biol..

[116] Van der Poll T., Opal S.M. (2008). Host-pathogen interactions in sepsis. Lancet Infect. Dis..

[117] Carson W.F., Cavassani K.A., Dou Y., Kunkel S.L. (2011). Epigenetic regulation of immune cell functions during post-septic immunosuppression. Epigenetics.

[118] Moreno P.R., Falk E., Palacios I.F., Newell J.B., Fuster V., Fallon J.T. (1994). Macrophage infiltration in acute coronary syndromes. Implications for plaque rupture. Circulation.

[119] Colin S., Chinetti-Gbaguidi G., Staels B. (2014). Macrophage phenotypes in atherosclerosis. Immunol. Rev..

[120] Van der Heijden C., Noz M.P., Joosten L.A.B., Netea M.G., Riksen N.P., Keating S.T. (2018). Epigenetics and Trained Immunity. Antioxid. Redox Signal..

[121] Steinbach E.C., Plevy S.E. (2014). The role of macrophages and dendritic cells in the initiation of inflammation in IBD. Inflamm. Bowel Dis..

[122] Moore K.J., Tabas I. (2011). Macrophages in the pathogenesis of atherosclerosis. Cell.

[123] Nagata S., Hanayama R., Kawane K. (2010). Autoimmunity and the clearance of dead cells. Cell.

[124] Nagata S. (2007). Autoimmune diseases caused by defects in clearing dead cells and nuclei expelled from erythroid precursors. Immunol. Rev..

[125] Janko C., Schorn C., Grossmayer G.E., Frey B., Herrmann M., Gaipl U.S., Munoz L.E. (2008). Inflammatory clearance of apoptotic remnants in systemic lupus erythematosus (SLE). Autoimmun. Rev..

[126] Kawane K., Ohtani M., Miwa K., Kizawa T., Kanbara Y., Yoshioka Y., Yoshikawa H., Nagata S. (2006). Chronic polyarthritis caused by mammalian DNA that escapes from degradation in macrophages. Nature.

[127] Bresnihan B. (1999). Pathogenesis of joint damage in rheumatoid arthritis. J. Rheumatol..

[128] Burmester G.R., Stuhlmuller B., Keyszer G., Kinne R.W. (1997). Mononuclear phagocytes and rheumatoid synovitis. Mastermind or workhorse in arthritis?. Arthritis Rheum..

[129] Gracie J.A., Forsey R.J., Chan W.L., Gilmour A., Leung B.P., Greer M.R., Kennedy K., Carter R., Wei X.Q., Xu D. (1999). A proinflammatory role for IL-18 in rheumatoid arthritis. J. Clin. Investig..

[130] Stuhlmuller B., Ungethum U., Scholze S., Martinez L., Backhaus M., Kraetsch H.G., Kinne R.W., Burmester G.R. (2000). Identification of known and novel genes in activated monocytes from patients with rheumatoid arthritis. Arthritis Rheum..

[131] Cas M.D., Roda G., Li F., Secundo F. (2020). Functional Lipids in Autoimmune Inflammatory Diseases. Int. J. Mol. Sci..

[132] Christ A., Bekkering S., Latz E., Riksen N.P. (2016). Long-term activation of the innate immune system in atherosclerosis. Semin. Immunol..

[133] Keating S.T., Plutzky J., El-Osta A. (2016). Epigenetic Changes in Diabetes and Cardiovascular Risk. Circ. Res..

[134] Keating S.T., Groh L., Thiem K., Bekkering S., Li Y., Matzaraki V., van der Heijden C., van Puffelen J.H., Lachmandas E., Jansen T. (2020). Rewiring of glucose metabolism defines trained immunity induced by oxidized low-density lipoprotein. J. Mol. Med..

[135] Stewart C.R., Stuart L.M., Wilkinson K., van Gils J.M., Deng J., Halle A., Rayner K.J., Boyer L., Zhong R., Frazier W.A. (2010). CD36 ligands promote sterile inflammation through assembly of a Toll-like receptor 4 and 6 heterodimer. Nat. Immunol..

[136] Duewell P., Kono H., Rayner K.J., Sirois C.M., Vladimer G., Bauernfeind F.G., Abela G.S., Franchi L., Nunez G., Schnurr M. (2010). NLRP3 inflammasomes are required for atherogenesis and activated by cholesterol crystals. Nature.

[137] Sheedy F.J., Grebe A., Rayner K.J., Kalantari P., Ramkhelawon B., Carpenter S.B., Becker C.E., Ediriweera H.N., Mullick A.E., Golenbock D.T. (2013). CD36 coordinates NLRP3 inflammasome activation by facilitating intracellular nucleation of soluble ligands into particulate ligands in sterile inflammation. Nat. Immunol..

[138] Netea M.G., Giamarellos-Bourboulis E.J., Dominguez-Andres J., Curtis N., van Crevel R., van de Veerdonk F.L., Bonten M. (2020). Trained Immunity: A Tool for Reducing Susceptibility to and the Severity of SARS-CoV-2 Infection. Cell.

[139] Erol A. (2020). Role of oxidized LDL-induced „trained macrophages” in the pathogenesis of COVID-19 and benefits of pioglitazone: A hypothesis. Diabetes Metab. Syndr..

[140] Theobald S.J., Simonis A., Georgomanolis T., Kreer C., Zehner M., Eisfeld H.S., Albert M.C., Chhen J., Motameny S., Erger F. (2021). Long-lived macrophage reprogramming drives spike protein-mediated inflammasome activation in COVID-19. EMBO Mol. Med..

[141] O’Neill L.A.J., Netea M.G. (2020). BCG-induced trained immunity: Can it offer protection against COVID-19?. Nat. Rev. Immunol..

[142] Reyes M., Filbin M.R., Bhattacharyya R.P., Billman K., Eisenhaure T., Hung D.T., Levy B.D., Baron R.M., Blainey P.C., Goldberg M.B. (2020). An immune-cell signature of bacterial sepsis. Nat. Med..

[143] Hotchkiss R.S., Monneret G., Payen D. (2013). Immunosuppression in sepsis: A novel understanding of the disorder and a new therapeutic approach. Lancet Infect. Dis..

[144] Bauer M., Giamarellos-Bourboulis E.J., Kortgen A., Moller E., Felsmann K., Cavaillon J.M., Guntinas-Lichius O., Rutschmann O., Ruryk A., Kohl M. (2016). A Transcriptomic Biomarker to Quantify Systemic Inflammation in Sepsis—A Prospective Multicenter Phase II Diagnostic Study. EBioMedicine.

[145] Starr M.E., Steele A.M., Cohen D.A., Saito H. (2016). Short-Term Dietary Restriction Rescues Mice From Lethal Abdominal Sepsis and Endotoxemia and Reduces the Inflammatory/Coagulant Potential of Adipose Tissue. Crit. Care Med..

[146] Chen X., Liu Y., Gao Y., Shou S., Chai Y. (2021). The roles of macrophage polarization in the host immune response to sepsis. Int. Immunopharmacol..

[147] Porta C., Rimoldi M., Raes G., Brys L., Ghezzi P., Di Liberto D., Dieli F., Ghisletti S., Natoli G., De Baetselier P. (2009). Tolerance and M2 (alternative) macrophage polarization are related processes orchestrated by p50 nuclear factor kappaB. Proc. Natl. Acad. Sci. USA.

[148] Seeley J.J., Ghosh S. (2017). Molecular mechanisms of innate memory and tolerance to LPS. J. Leukoc. Biol..

[149] Parihar A., Eubank T.D., Doseff A.I. (2010). Monocytes and macrophages regulate immunity through dynamic networks of survival and cell death. J. Innate Immun..

[150] Otto G.P., Sossdorf M., Claus R.A., Rodel J., Menge K., Reinhart K., Bauer M., Riedemann N.C. (2011). The late phase of sepsis is characterized by an increased microbiological burden and death rate. Crit. Care.

[151] Wang T.S., Deng J.C. (2008). Molecular and cellular aspects of sepsis-induced immunosuppression. J. Mol. Med..

[152] Cao C., Yu M., Chai Y. (2019). Pathological alteration and therapeutic implications of sepsis-induced immune cell apoptosis. Cell Death Dis..

[153] Cheng S.C., Scicluna B.P., Arts R.J., Gresnigt M.S., Lachmandas E., Giamarellos-Bourboulis E.J., Kox M., Manjeri G.R., Wagenaars J.A., Cremer O.L. (2016). Broad defects in the energy metabolism of leukocytes underlie immunoparalysis in sepsis. Nat. Immunol..

[154] Quintin J., Saeed S., Martens J.H.A., Giamarellos-Bourboulis E.J., Ifrim D.C., Logie C., Jacobs L., Jansen T., Kullberg B.J., Wijmenga C. (2012). Candida albicans infection affords protection against reinfection via functional reprogramming of monocytes. Cell Host Microbe.

[155] Chen K., Geng S., Yuan R., Diao N., Upchurch Z., Li L. (2015). Super-low dose endotoxin pre-conditioning exacerbates sepsis mortality. EBioMedicine.

[156] Noy R., Pollard J.W. (2014). Tumor-associated macrophages: From mechanisms to therapy. Immunity.

[157] Franklin R.A., Liao W., Sarkar A., Kim M.V., Bivona M.R., Liu K., Pamer E.G., Li M.O. (2014). The cellular and molecular origin of tumor-associated macrophages. Science.

[158] Ostuni R., Kratochvill F., Murray P.J., Natoli G. (2015). Macrophages and cancer: From mechanisms to therapeutic implications. Trends Immunol..

[159] Rackov G., Hernandez-Jimenez E., Shokri R., Carmona-Rodriguez L., Manes S., Alvarez-Mon M., Lopez-Collazo E., Martinez A.C., Balomenos D. (2016). p21 mediates macrophage reprogramming through regulation of p50-p50 NF-kappaB and IFN-beta. J. Clin. Investig..

[160] Mu Q., Najafi M. (2021). Modulation of the tumor microenvironment (TME) by melatonin. Eur. J. Pharmacol..

